# The role of unique color changes and singletons in attention capture

**DOI:** 10.3758/s13414-016-1139-y

**Published:** 2016-05-20

**Authors:** Adrian von Mühlenen, Markus Conci

**Affiliations:** 1 Department of Psychology, University of Warwick, Coventry, CV4 7AL UK; 2 Ludwig-Maximilians-University, Munich, Germany

**Keywords:** Attentional capture, Visual search, Reaction time methods

## Abstract

Previous studies have shown that a sudden color change is typically less salient in capturing attention than the onset of a new object. Von Mühlenen, Rempel, and Enns (Psychological Science 16: 979-986, 2005) showed that a color change can capture attention as effectively as the onset of a new object given that it occurs during a period of temporal calm, where no other display changes happen. The current study presents a series of experiments that further investigate the conditions under which a change in color captures attention, by disentangling the change signal from the onset of a singleton. The results show that the item changing color receives attentional priority irrespective of whether this change goes along with the appearance of a singleton or not.

## Introduction

When confronted with the task of finding a certain object among other objects, our ability to identify the target quickly depends on a number of factors. It depends on our knowledge about the nature of the target, which can be used to guide attention more efficiently to locations that are more likely to contain the target (e.g., Kim & Cave, 1995). For example, when picking up someone from the airport, our knowledge about this person (e.g., a long white beard) will likely guide our attention to any person with white hair. But search efficiency also depends on how easily we can filter out things or events that might be noticeable, but not relevant for the current task. These might include certain static features, like a distinctive color (e.g., red hair), or they might include certain dynamic events, like appearing objects, moving objects, or changing objects (e.g., a change in color). This ability to ignore certain features, objects, or events has been the subject of numerous studies conducted in the last 40 years under the heading “attention capture,” where an object or event is said to capture attention when it cannot be ignored.

Everyday experience may suggest that any salient change in the visual field captures our attention, as it might signal something requiring our immediate attention. However, research in the psychophysical laboratory has shown that this is not the case. We are, as a matter of fact, very effective in ignoring sudden changes when they are irrelevant for, or diverting away from, what one is currently doing. This has been shown for changes in color (e.g., Awh, Belopolsky, & Theeuwes, 2012; Jonides & Yantis, 1988; Theeuwes, 1990, 1995), changes in luminance (Enns, Austen, Di Lollo, Rauschenberger, & Yantis, 2001; Jonides & Yantis, 1988), and changes in motion (Hillstrom & Yantis, 1994; Yantis & Egeth, 1999). The only exceptions revealing strong attentional capture were changes that included the onset of a new object (Hillstrom & Yantis, 1994; Jonides & Yantis, 1988). It was argued that simple changes of features in existing objects are far too common in our natural environment to be informative of behaviorally urgent events, and only the appearance of a new object is potentially important to our survival (Hillstrom & Yantis, 1994; Jonides & Yantis, 1988). Others have argued that onsets are special because they require the creation of a new representation (a so called “object file”), a process known to involve attentional resources (cf. Kahneman, Treisman & Gibbs, 1992).

**Fig. 1:**
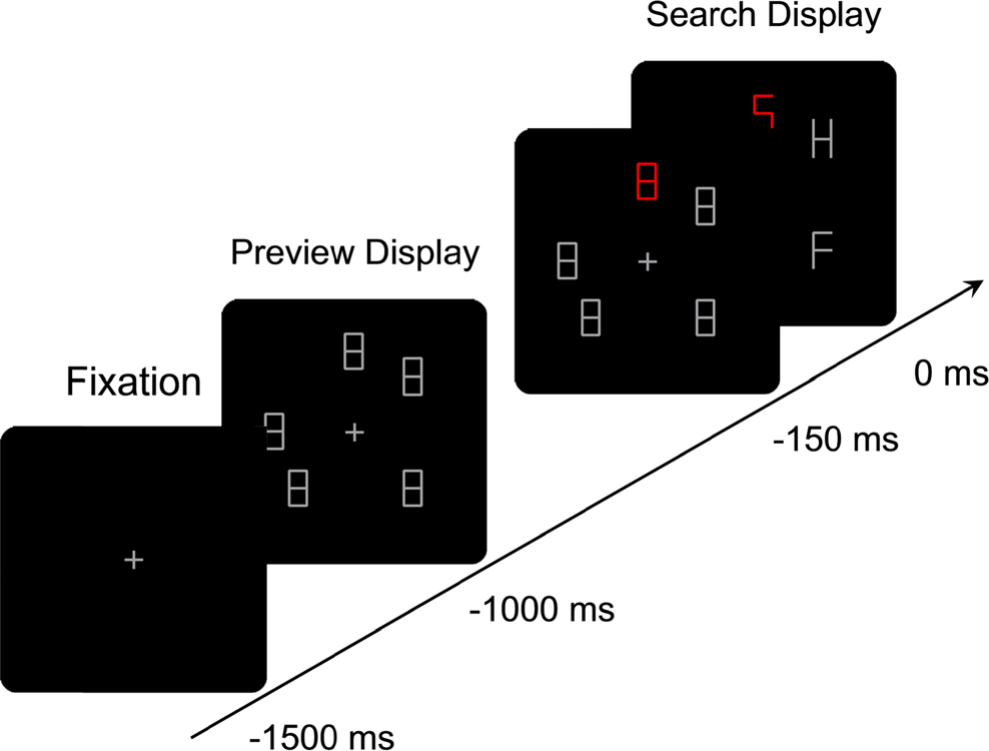
Schematic representation of the sequence of events in Experiment 1 with an example of a five-item display. The search displays shows a unique distractor “S”, a non-unique target “H”, and a non-unique distractor “F”

A study by von Mühlenen, Rempel, and Enns (2005) pointed to another temporal factor that is critical to the capture of attention, over and above any of these considerations. They used a variant of Todd & Van Gelder (1979) placeholder search paradigm, where a search display is preceded by a preview display consisting of figure-eight placeholders, and after 1 s some line segments of each figure eight are deleted to reveal the letters of the search display (see Fig. 1 for an example display). Von Mühlenen et al. systematically varied the timing of events, where a change could occur either during the preview, simultaneously with the preview-search transition, or during the search. The event included either a change in color, a change in motion (i.e., a motion onset or offset), or the onset of a new letter, and it occurred either with the target or with one of the distractor letters. Attention capture was assessed through the search slopes (i.e., the slope of the response time, RT, plotted as a function of the number of letters in the display), which were calculated separately for when the target changed, compared to when one of the distractors changed. A reduced search slope for changing targets (relative to changing distractors) would indicate that the changed item captured attention. They showed that changes in color or in motion (i.e., motion onset or motion offset) could be as effective as an onset in capturing attention, provided that it occurred during a period of temporal calm. When the same change occurred simultaneously with the transition from preview to search display then it failed to capture attention. Von Mühlenen et al. argued that the change ceased to capture attention because it was concealed by the other changes (removal of line segments) occurring during display transition. This finding was not necessarily contradictory to previous findings; it rather offered an extension of the existing accounts, highlighting the importance of temporal factors in the study of attentional capture.

There are only a few other studies where color changes were shown to capture attention. For example, Turatto and Galfano (2000, 2001) reported that a color change can capture attention when participants are relatively inexperienced in visual search tasks. In another study a color change captured attention when it was unexpected and/or surprising (Horstmann, 2002, 2015). However, these studies used a different measure of attentional capture, one not based on the systematic variation of display size (see Rauschenberger, 2003, for a critical discussion of measures not based on the search slope ratio, but see also Turatto, Galfano, Gardini, & Mascetti, 2004, for an opposite view).

Another important factor shown to influence attention capture has been the ability to setup top-down control. In one study by Lu and Zhou (2005) color changes were shown to capture attention, but only when the color-to-stimuli assignment was unpredictable from trial to trial. This was taken up by von Mühlenen and Conci (2009b), who showed that ignoring the color change depends critically on the ability to establish some form of top-down control. They suggested that this top-down control included a facilitatory process that was enhancing all non-changed items. Other top-down factors determining whether a color change captures attention include task demand (e.g., Folk, Remington, & Wright, 1994; Schmidt & Schmidt, 2010; Scholl, 2000; Theeuwes & Burger, 1998). Note that some of these studies used a different methodology to assess capture effects and hence they won't be further discussed here. For a brief comparison of paradigms commonly used to assess capture see Simons (2000).

A few studies using the irrelevant feature paradigm found weak capture effects with color changes (e.g., von Mühlenen et al., 2005; Todd & Kramer, 1994). Todd and Kramer argued that participants might adopt a top-down search strategy where these irrelevant features serve as landmarks from which to begin search. However, because participants had neither an incentive nor an advantage in using such a strategy, we would assume that they quickly learn to ignore an irrelevant color feature. In a re-analysis of four color-capture experiments (N = 46), we showed that both color changes and onsets initially (i.e., in the first 90 trials) do capture attention, but thereafter the effect disappears for color changes, whereas onsets continue to capture attention (von Mühlenen & Conci, 2009a).

With motion onset, Abrams and Christ (2003) found – in contrast to von Mühlenen et al. (2005) – capture for motion onset even when it co-occurred with display transition. However, there were a number of notable differences between the two studies in terms of design and measures for attentional capture. Most critically, von Mühlenen et al. used relatively smooth motion (85 Hz), whereas Abrams and Christ (2003) used rather jerky motion (15 Hz). Indeed Sunny and von Mühlenen (2011) were able to show that motion onset capture only occurred with the form of jerky motion, but not with smooth motion as used by von Mühlenen et al. (2005; see also Sunny & von Mühlenen, 2014).

In order to account for temporal factors in attention capture, von Mühlenen et al. (2005) proposed their unique-event account, according to which an event captures attention when it is temporally unique. However, what exactly captures attention is not clear. It could be either the local change signal that goes along with the color change, but it could also be the appearance of a color singleton that captures attention. This study further explores the conditions under which a unique color change captures attention, by disentangling the color change signal from the color singleton onset.

The present study presents five experiments where a color change occurred 150 ms before display transition, a condition which showed a strong capture effect in von Mühlenen et al.’s study. In the first three experiments a color change happened in one single item. In Experiment 1 one of the gray placeholders changed its color to red (single change, singleton onset). In Experiment 2 a red singleton changed its color to gray (single change, singleton offset). In Experiment 3 placeholders all had different colors, and one item changed color (single change, no singleton). In the last two experiments the color change occurred in all items. In Experiment 4a all placeholders were gray, one changed to red, and the others to green. In Experiment 4b one placeholder was red, the others were green, and they all changed to gray.

## Experiment 1

In the first experiment one of the gray figure-eight placeholders changed color to red 150 ms before the search letters were revealed. The procedure is very similar to von Mühlenen et al. (2005; Experiment 1, -150 ms condition), and we expected to find a significant attention capture effect for this temporally unique change in color.

### Method

#### Participants

Ten participants (four male, mean age = 26.3 years) from the Ludwig-Maximilians-University Munich participated in the experiment receiving payment of 8 Euros per hour. All of them reported normal or corrected-to-normal vision and were naïve to the purpose of the experiment.

#### Apparatus and stimuli

The experiment was controlled by an IBM-PC compatible computer using Matlab routines and Psychophysics Toolbox extensions (Brainard, 1997; Pelli, 1997). The stimuli were presented on a 17-in. monitor, at a resolution of 1,024 × 768 pixels, and participants’ responses were recorded via the mouse keys. Prior to the experiment, stimulus luminance was measured using a Konica Minolta CS-200/LS-100 luminance meter to ensure that all presented display items were equiluminant. Stimuli consisted of a fixation cross, placeholders, and letters, presented either in gray (luminance 8.5 cd/m^2^) or in red (8.5 cd/m^2^) drawn on black background (0.02 cd/m^2^). The fixation cross had a size of 0.6° of visual angle, the figure-eight placeholders and the letters subtended 1° by 2.0°. The placeholders had the shape of an “8” and were made of three horizontal and four vertical line segments (length 1.0°, thickness 0.13°). The letters H, U, E, P, S, C, F, and L, were made by removing the corresponding line segments from the figure eight (e.g., the top and bottom horizontal line segment of the “8” was removed to reveal the letter H). Stimuli were randomly distributed among eight locations, evenly spaced on an imaginary circle (5.3° in radius) around the fixation cross. The target letter was either the letter H or U and the distractor letters were randomly chosen from the other letters, with the constraint that a letter was presented only once on a given trial.

#### Procedure

A typical trial sequence is shown in Fig. 1. A trial started with a fixation cross presented for 500 ms, followed by the preview display, which (depending on display size) contained either three, five, or seven placeholders presented for 1,000 ms. During the first 850 ms all placeholders were gray before one of them changed its color to red for 150 ms. After that all figure eights changed into letters and stayed on-screen until a response was made. Participants were instructed to search for the target letter U or H and to respond with the mouse buttons. Half of the participants used the left mouse button for H and right button for U, and vice versa for the other half. Response times were measured from the onset of the letter-search display. Participants were instructed to respond to the target as fast as they could whilst trying to keep errors below 5 %. In the instance of wrong responses visual feedback was given in the form of an alerting sign "–" presented at the center of the screen for 1 s. Participants were also told that the position of the uniquely colored stimulus was uninformative with respect to the location of the target. The next trial started after an interval of 1 s. Each participant completed 25 practice trials followed by 360 experimental trials. The experimental trials were divided into six blocks of 60 trials each, with short breaks between blocks.

#### Design

The experiment systematically varied three factors: target identity (H or U), display size (three, five, or seven items), and target type (unique, non-unique). For every display size, the target was equally likely to be the unique or one of the non-unique items. Thus for display size three there were 72 (24 unique and 48 non-unique) trials, for display size five there were 120 (24 unique and 96 non-unique) trials, and for display size seven there were 168 (24 unique and 144 non-unique) trials. All possible factor combinations were presented in random order. In the analysis, target identity was not further considered.

#### Analysis

Attention capture is indexed by the relative improvement in RT slopes for a changing target compared to a changing distractor. This is based on the assumption that when a unique change draws attention to itself, it will speed up the search process if it occurs at the target and slow down search if it occurs at a distractor. A non-unique target to unique target slope ratio of 1:1 means that the unique change had no differential effect on the search efficiency and, thus, no effect on attention (e.g., Folk & Annett, 1994; Franconeri & Simons, 2003; Jonides & Yantis, 1988; Todd & Kramer, 1994; von Mühlenen, Rempel, & Enns, 2005; Yantis & Egeth, 1999). In the current study we will report logarithmic slope ratios. This has the advantage that a ratio of 1:1 becomes zero, which better represents the absence of an effect on attention. It also has the advantage that a positive value represents an increased processing priority (i.e., capture) and a negative value a reduced processing priority (i.e., inhibition). Finally extreme values (e.g., large values due to a target slope close to zero) have less weight.

### Results and discussion

#### Errors

Mean error percentages were calculated separately for each participant and factor combination, and then submitted to a 2 × 3 repeated measures ANOVA with the factors target type (unique or non-unique) and display size (three, five, or seven items). The ANOVA revealed a marginally significant effect for target type, F(1,9) = 4.43, p = .065, due to slightly more errors for when the target was non-unique than when it was unique (5.4 % vs. 3.9 %, respectively). Because RTs show a similar effect, we can safely assume that they are not confounded by speed-accuracy tradeoffs. Overall participants made 4.6 % errors, complying well with the instruction of keeping errors below 5 % (see Table 1).

**Table 1:** Mean error percentages and mean response times (RTs) and slopes (in ms per item) in Experiment 1

	Error (%)			RT (ms)			
	Display size			Display size			
Target type	3	5	7	3	5	7	Slope
Non-unique	7.5	4.3	4.4	649	701	773	31.1
Unique	3.6	4.6	3.4	595	651	652	14.3

#### Response times (RTs)

**Fig. 2:**
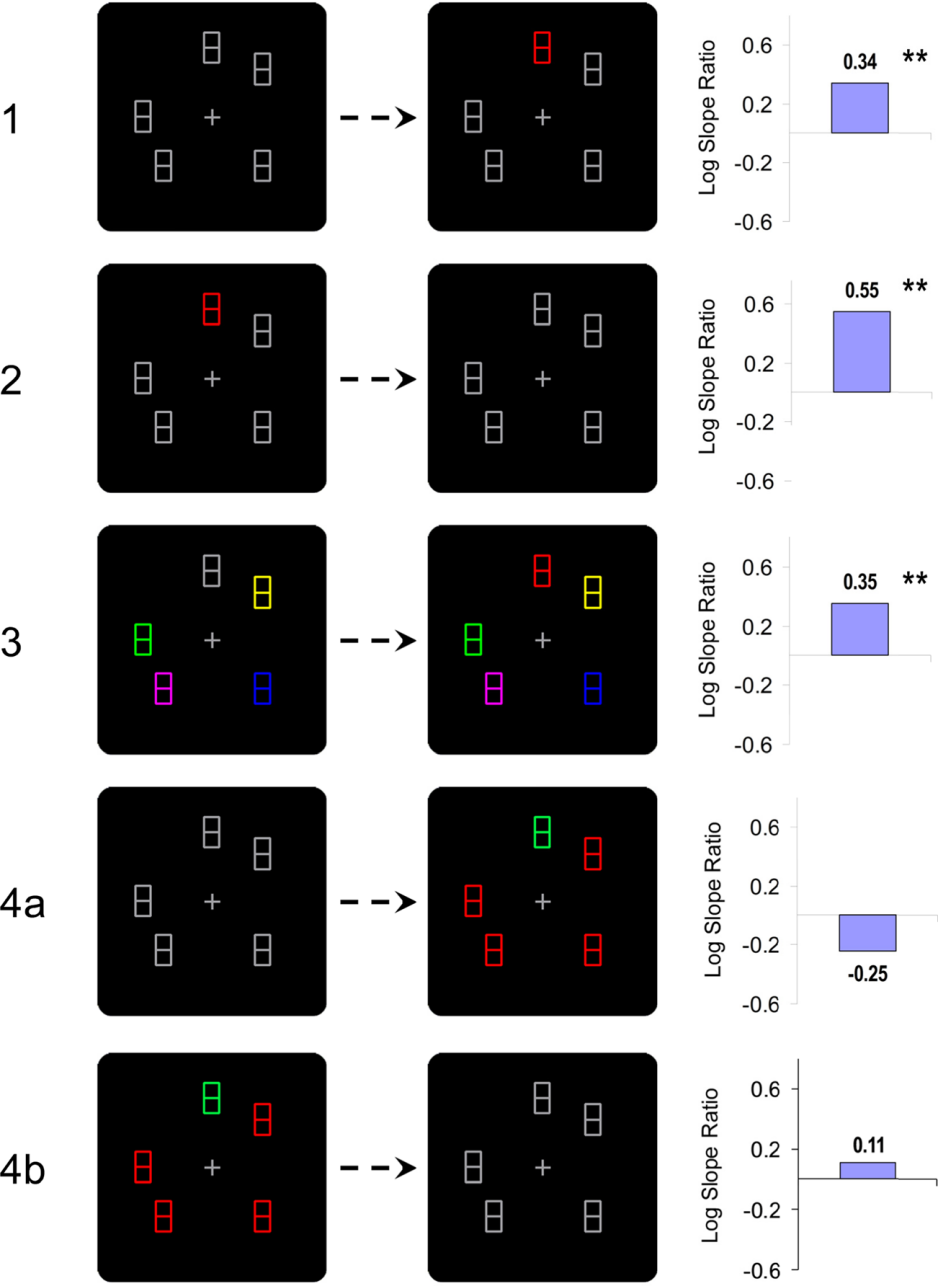
Color changes and corresponding capture index (log slope ratio) for Experiments 1–4. The stars next to the capture index indicate the significance of the interaction in the corresponding response time (RT) ANOVA with the factors display size and target type

Median RTs were calculated for each participant and factor combination, excluding errors. The overall means, averaged across participants, and the corresponding search slopes (in ms per item) are shown in Table 1. The individual median RTs were submitted to a 2 × 3 ANOVA with the factors target type (unique or non-unique) and display size (three, five, or seven items). Both main effects, for display size, F(2,18) = 19.28, p < .001, and for target type, F(1, 9) = 15.83, p = .003, were highly significant: RTs increased with display size on average by 22.7 ms/item (from 622, 676, to 713 ms, respectively), and search was 75 ms faster when the target was the unique item compared to when it was the non-unique items. The two-way interaction was also significant, F(2,18) = 4.22, p = .031, due to faster search slopes with unique than with non-unique targets (14 vs. 31 ms/item, respectively). Moreover, as can be seen from Table 1, the search slope difference was more pronounced between display size five and seven (0:36 ms/item, respectively) than between display size three and five (28:26 ms/item, respectively). A summary of Experiment 1, illustrating the color change and the resulting attention capture index, is given in Fig. 2.

Overall the results of Experiment 1 replicate von Mühlenen et al.’s (2005) finding of significant capture for a color change that was temporally unique. Although the unique to non-unique slope ratio in the current study (14:31 ms/item) is a bit smaller than in von Mühlenen et al.’s experiment (12:34 ms/item), the overall slope reduction looks very similar. Moreover, in von Mühlenen et al. the search slope difference was also more pronounced between display size five and seven (0:45 ms/item, respectively) than between display size three and five (23:23 ms/item, respectively). We can only speculate that this might be linked to the way saliency is calculated, such that with more items in the display, the salience of the changing item is increased. Experiment 1 thus provides further support for the idea that capture can occur by means of a unique color change when the change occurs 150 ms before display transition.

According to the unique-event account (von Mühlenen et al., 2005), attention capture critically depends on a local temporally unique change. They showed that when there are other changes occurring simultaneously in the display, then the same local change stopped capturing attention, presumably because it was no longer unique. In their study these simultaneous changes could include the onset of other items or the removal of figure-eight segments revealing the search letters. The subsequent experiments will look at the effect of other forms of color changes, and at the role of the onset or offset of a color singleton.

## Experiment 2

In Experiment 1 one of the gray items changed its color to red at a time where nothing else happened. It can be argued that this change comes along with changes at two levels: At a local level there is a change in color (gray-red transition) at the location of the item, whereas at a more global level there is a change in terms of appearance of a new color singleton (red-singleton onset). According to the unique-event account, capture would hinge rather on the local color transition than on the global singleton onset. In Experiment 2 we subjected this prediction to a first test by using the same color transition (but in reversed order), and by removing the color singleton from the search display. It was predicted that if having a singleton onset in the search phase was the critical factor, then there should be no more capture effect.

### Method

Ten participants (four male, mean age = 28.8 years) from the Ludwig-Maximilians-University Munich participated in the experiment receiving payment of 8 Euros per hour. All reported normal or corrected-to-normal vision, they had not participated in the previous experiment, and they were naïve to the purpose of the experiment.

Apparatus, stimuli, procedure, and design were the same as in Experiment 1, with the only difference that the color change of the unique item was now the other way round, from red to gray. Thus, there was first one red placeholder amongst gray placeholders, which after 850 ms changed its color to the same gray as the other placeholders (see Fig. 2).

### Results and discussion

#### Errors

The averaged error percentages are given in Table 2. A 2x3 ANOVA with the factors target type and display size revealed no significant effects (all F<1), suggesting that RTs are not confounded by speed-accuracy tradeoffs.

**Table 2:** Mean error percentages and mean response times (RTs) and slopes (in ms per item) in Experiment 2

	Error (%)			RT (ms)			
	Display size			Display size			
Target type	3	5	7	3	5	7	Slope
Non-unique	2.5	2.3	2.1	598	665	743	36.2
Unique	3.1	3.3	1.3	605	664	646	10.2

#### RTs

The averaged median RTs and corresponding search slopes are given in Table 2. A 2 × 3 ANOVA with the factors target type and display size revealed a significant main effect for display size, F(2,18) = 33.60, p < .001, and a marginally significant effect for target type, F(1, 9) = 3.99, p = .077. The interaction was also significant, F(2,18) = 9.91, p = .001, which was due to faster search slopes with unique than with non-unique targets (10 vs. 36 ms/item, respectively). Again, as can be seen from Table 2, the search slope difference was more pronounced between five and seven (-9:39 ms/item, respectively) than between display size three and five (33:29 ms/item, respectively).

Figure 2 suggests that attention capture was stronger in Experiment 2 than in Experiment 1; however, in a mixed-design ANOVA the corresponding 3-way interaction was not significant (p = .62). Nevertheless, the absence of a color-singleton onset did not lead to the absence of a capture effect. This suggests that having a singleton in the search display is not a critical component for obtaining a capture effect. It supports the view that the local color transition is the critical component to obtain a capture effect. However, one could argue that the presence of a color singleton during the first (850 ms) preview period was sufficient for capture. This possibility was further explored in Experiment 3.

## Experiment 3

In the first two experiments, there was always a color singleton present either during the search phase (Experiment 1) or during the preview phase (Experiment 2). In the next experiment we wanted to remove the color singleton from both (preview and search) displays. However, it is, in principle, not possible to get from a uniformly colored preview display to a uniformly colored search display when only one item is allowed to change color. So in order to remove the singleton from both displays we used displays with multiple colors, where every item had a different color (thus none of them was singled out by its color). In other words, we had displays with no singleton but with one single change. Again it was predicted that if it was critical for capture to have a singleton in either the preview or the search phase, then there should be no capture effect in Experiment 3.

### Method

Ten participants (five male, mean age = 27.0 years) from the Ludwig-Maximilians-University Munich participated in the experiment. They reported normal or corrected to normal vision and were naïve to the purpose of the experiment.

Apparatus, stimuli, procedure, and design were the same as before, except that now every placeholder had a different color – randomly chosen from eight possible (equiluminant, 6.9 cd/m2) colors: yellow, red, green, magenta, purple, blue, gray, and brown. Then after 850 ms one of these placeholders changed color randomly to one of the remaining colors (see Fig. 2).

### Results and discussion

#### Errors

The averaged error percentages are given in Table 3. A 2x3 ANOVA with the factors target type and display size revealed no significant effects (all F<1), suggesting that RTs are not confounded by speed-accuracy tradeoffs.

**Table 3:** Mean error percentages and mean response times (RTs) and slopes (in ms per item) in Experiment 3

	Error (%)			RT (ms)			
	Display size			Display size			
Target type	3	5	7	3	5	7	Slope
Non-unique	2.0	1.7	1.9	607	656	716	27.4
Unique	3.5	2.4	2.0	602	670	651	12.1

#### RTs

The averaged median RTs and corresponding search slopes are given in Table 3. A 2 × 3 ANOVA with the factors target type and display size revealed a significant main effects for display size, F(2,18) = 29.48, p < .001, and target type, F(1, 9) = 10.93, p = .009, and a significant interaction, F(2,18) = 8.35, p = .002, which was due to faster search slopes with unique than with non-unique targets (12 vs. 27 ms/item, respectively). Again, as can be seen from Table 3, the search slope difference was more pronounced between display size five and seven (-10:30 ms/item, respectively) than between display size three and five (34:25 ms/item, respectively).

Figure 2 shows that the capture effect in this experiment was numerically very similar to the one in Experiment 1. Thus, the absence of a color-singleton in both preview and search display did not lead to the absence of a capture effect. It suggests that having a singleton is not essential, but having a local color transition seems to be the critical component to obtain a capture effect. In this experiment, we were removing the singleton information from the display while keeping the local transition information. In the next experiment we were doing the opposite, removing the unique local transition information, while keeping the singleton information.

## Experiment 4

In Experiment 4 the single local color transition was replaced by multiple color changes occurring in each item. The experiment tested two conditions, which were following the same manipulation used in Experiments 1 and 2. Experiment 4a used a condition equivalent to Experiment 1, starting with a preview of uniform gray placeholders. However, after 850 ms *all* items changed color – one item to green and the other items to red. Thus the search display contained one color singleton, but at the same time all items revealed a local color transition. Experiment 4b was the condition equivalent to Experiment 2. As in Experiment 4a, all items revealed a local color transition, but the singleton was now present in the preview display. Hence, the placeholder display started with one green amongst red items, and after 850 ms all of them changed to gray. In Experiments 1–3 we had argued that attention capture is primarily driven by a single local color change. We therefore predicted for both Experiments 4a and 4b, that having a color singleton on its own without a local color transition was not sufficient to capture attention.

### Method

Twenty participants (seven male, mean age = 24.8 years) from the Ludwig-Maximilians-University Munich took part in the experiment. They reported normal or corrected-to-normal vision and were naïve to the purpose of the experiment. Ten participants took part in Experiment 4a and ten participants in Experiment 4b.

Apparatus, stimuli, procedure, and design were similar to Experiments 1 and 2. In Experiment 4a all placeholders started out in gray. Subsequently one placeholder changed color from gray to green and the others from gray to red (see Fig. 2). In Experiment 4b we used exactly the same stimuli as in Experiment 4a, but the changes occurred in reversed order, that is, placeholders changed from red and green to gray (see Fig. 2).

### Results and discussion

#### Errors

The averaged error percentages are given in Table 4. A 2x2x3 mixed-design ANOVA with the within-subject factors target type and display size and the between-subject factor singleton (at preview, during search) revealed a marginally significant effect for target type, F(1,18) = 4.39, p = .050, due to slightly less errors for when the target was non-unique than when it was unique (2.2 % vs. 2.9 %, respectively), and a significant interaction between display size and singleton, F(2,36) = 5.82, p = .006. In Experiment 4a, there were less errors with display size three, compared to display size five and seven (2.1 % vs. 3.6 % and 3.1 %, respectively), whereas in Experiment 4b, there were more errors with display size three (3.1 % vs. 2.0 % and 1.8 %, respectively). Because none of the interactions involving target type and display size were significant, it seems unlikely that RT results are confounded by speed-accuracy tradeoffs.

**Table 4:** Mean error percentages and mean response times (RTs) and slopes (in ms per item) in Experiment 4a and 4b

	Error (%)			RT (ms)			
	Display size			Display size			
Target type	3	5	7	3	5	7	Slope
Experiment 4a							
Non-unique	1.6	2.8	2.7	581	624	671	22.6
Unique	2.5	4.3	3.4	607	663	766	39.9
Experiment 4b							
Non-unique	3.1	1.4	1.8	603	652	690	21.8
Unique	3.1	2.5	1.8	609	677	677	17.1

#### RTs

The averaged median RTs and corresponding search slopes are given in Table 4. A 2 × 2 × 3 mixed-design ANOVA with the within-subject factors target type and display size and the between-subject factor singleton (at preview, during search) revealed significant main effects for target type, F(1, 9) = 6.18, p = .023, and for display size, F(2,36) = 54.53, p < .001, and a significant interaction effect between display size and singleton, F(2,36) = 4.82, p = .013. RTs were somewhat faster with unique than with non-unique targets (637 vs. 666 ms, respectively). RTs increased with display size, and this search slope was less pronounced in Experiment 4a (with the singleton at preview) than in Experiment 4b (with the singleton during search, 31.2 vs. 19.5, respectively). There was also a marginally significant interaction between target type and singleton, F(1,18) = 3.99, p = .061, and a marginally significant 3-way interaction, F(2,36) = 3.33, p = .057. As can be seen in Table 4, in Experiment 4a the search slope appears to be shallower for unique than for non-unique targets. However, a separate ANOVA including only the data from Experiment 4a showed no significant interaction between target type and display size, F(2,18) = 2.37, p = .12. Hence the slope increase from 23 ms/item (for non-unique targets) to 40 ms/item (for unique targets), which would be indicating inhibition, was statistically not reliable.

Finally, in order to compare the capture effects in Experiment 4a and 4b with the corresponding conditions in Experiment 1 and 2, an overall 4-way mixed design ANOVA was calculated with the additional between-subject factor changed items (1, n). Of the interactions involving target type and display size (which are indicators for capture), both 3-way interactions were significant, but not the 4-way interaction (F<1). The changed items x target type x display size interaction, F(2, 72) = 9.07 p < .001, indicates that the capture effect is significantly reduced in Experiment 4a and 4b (n items change) in comparison to Experiments 1 and 2 (only one item changes). In fact, the capture effect is either so small that it is statistically not significant (Experiment 4b, p = .28), or it is in the opposite direction, even though this indication of an inhibitory effect did not reach statistical significance (Experiment 4a, p = .12). The singleton x target type x display size interaction shows that the capture effect is significantly larger in Experiment 2 and 4b in comparison to Experiment 1 and 4a. This result is in line with von Mühlenen et al.’s (2005) unique-event account, according to which a unique change is a critical precondition to obtain a capture effect. Following this logic, a unique item, which is presented during a period were no other changes occur captures attention more than a unique item which is presented during the placeholder-search display transition.

## General discussion

We have presented four experiments where a color change occurred 150 ms before display transition, a condition which showed a strong capture effect in von Mühlenen et al.’s (2005) study. In Experiment 1 one of the gray placeholders changed its color to red, which led to a robust capture effect similar in magnitude to the one reported in von Mühlenen et al.’s (2005) corresponding condition in their first experiment. In Experiment 2 the sequence of events was reversed, with a red singleton changing to gray, which led to an even stronger capture effect than in the first experiment. In Experiment 3 all placeholders had different colors, but only one item changed its color. Despite the absence of a color-singleton in both preview and search display there was a significant capture effect for the color-changing item. In the final experiment color changes occurred in all items, but there was a singleton in either the preview display (Experiment 4a) or in the search display (Experiment 4b). In this experiment there was now no capture effect for the item containing the color singleton.

These findings suggest that having a singleton in the search display is neither necessary nor sufficient to obtain a capture effect. They provide strong support for the view that a unique local color transition is on its own sufficient for the occurrence of a robust capture effect. On the whole these findings are in line with the basic idea behind the unique-event account, according to which any sudden change is capable of capturing attention, as long as it is temporally unique. This account corresponds to a purely bottom-up model of attention capture, which suggest that capture is triggered by an increased saliency signal that accompanies the color change (Theeuwes, 2010). It builds on the idea that the local color change produces a transient salience signal that is only briefly represented in the visual system. It is in line with recent studies that have shown that such salience signals can be very short-lived (e.g., Donk & Soesman, 2010; Donk, & van Zoest, 2008). Re-entrant processes take over after the initial feed forward sweep, and the identity of the letters could be actively prioritized over its onset status (Di Lollo, Enns, & Rensink, 2000). We suggest that such color changes automatically produce an attend-to-me signal, irrespective of top-down control settings, but that this can be overridden by an active suppression process when other changes occur in the display simultaneously or close in time (Sawaki & Luck, 2010). As such, the unique-event account provides a useful framework that can account for a wide range of findings.
